# Post-treatment renal function deterioration following radiation therapy: implications for SABR in primary renal cell cancer

**DOI:** 10.3389/fonc.2026.1868006

**Published:** 2026-06-23

**Authors:** Marthe Sophie Kilian, Laura Anna Fischer, Jona Bensberg, Lisa-Antonia von Diest, Carla Marie Zwerenz, Mahalia Z. Anczykowski, Stephanie Bendrich, Manuel Guhlich, Martin Leu, Leif Hendrik Dröge, Tibor I. Kesztyüs, Annemarie Uhlig, Stefan Rieken, Rami A. El Shafie

**Affiliations:** 1 Department of Radiation Oncology, University Medical Center Göttingen, Göttingen, Germany; 2 Medical Data Integration Center (MeDIC), University Medical Center Göttingen, Göttingen, Germany; 3 Department of Urology, University Medical Center Göttingen, Göttingen, Germany; 4 Department of Urology, University Hospital Tübingen, Tübingen, Germany

**Keywords:** chronic kidney disease, dose-response relationship, kidney dose constraints, nephron-sparing therapy, primary renal cell carcinoma, radiation-associated kidney injury, renal function decline, stereotactic ablative radiotherapy

## Abstract

**Background:**

Definitive renal stereotactic ablative radiotherapy (SABR) is gaining momentum, with promising data showing excellent local control and short-term renal function comparable to surgery. However, data on late radiation-associated kidney injury and renal safety remain limited. We therefore assessed post-treatment renal outcomes and dose-response after kidney irradiation to inform nephron-sparing SABR for primary renal cell carcinoma (pRCC).

**Methods:**

We conducted a retrospective cohort study of trunk radiotherapy patients at a tertiary university cancer center, with incidental kidney exposure (EQD2 ≥10 Gy) and longitudinal renal function follow-up. Of 731 screened patients, 232 met inclusion criteria. Chronic-kidney-disease (CKD) deterioration was analyzed against renal dose using multivariable Cox, cut-off, and spline analyses.

**Results:**

During median follow-up of 170 days (IQR 60-541), CKD worsening occurred in 46.98% of patients, at a median of 173 days (IQR 55-423). Mean and maximum kidney doses (EQD2) were 14.2 Gy (SD 4.68) and 14.6 Gy (SD 4.86), respectively. Higher renal dose independently predicted renal function decline (HR 1.04/Gy; p=0.028 for mean; p=0.014 for max). Female sex was a risk factor (HR 2.08; p<0.001). Cut-offs were 18.7 Gy (mean) and 19.0 Gy (max), with monotonic dose-response. Retrospective design limits causal inference; constraints remain exploratory; validation required.

**Conclusions:**

Within low-to-moderate dose ranges to non-tumorous kidney tissue, irradiation was associated with a dose-dependent post-treatment decline in renal function, while severe toxicity was rare. Nephron-sparing SABR is a safe treatment option for pRCC, informing risk-adapted dose constraint refinement to minimize late injury.

## 1 Introduction

Renal cell carcinoma (RCC) is the third most common malignancy in urology, with incidence rates of 10–12 per 100,000 person-years in high-income countries (1–3). It’s mainly treated surgically, with partial nephrectomy (PN) for localised cT1 disease and radical nephrectomy (RN) for cT2-T3 or complex tumors (4). While oncologically effective, both procedures carry a risk of renal function decline (RFD) (5, 6). After PN, ischemia time contributes to early injury, with acute kidney injury occurring in 25–40% of patients (7–9). Long-term outcomes, including progression to chronic kidney disease (CKD) in 20-30% within one year, are influenced by baseline renal function and preserved parenchyma (5–8). CKD rates are higher after RN than PN (56% vs 44%) and 29% of PN patients with CKD stage 1–2 developed CKD stage ≥3 (10, 11). Radiotherapy (RT) has been considered ineffective in RCC, leaving medically inoperable patients with limited treatment options (12–15). This stems from RCC’s highly vascularised biology and its dependence on endothelial apoptosis for tumor control (14, 16). Conventional fractionated RT inadequately targets these mechanisms, resulting in poor responses (12, 17). In contrast, high-dose-per-fraction techniques such as stereotactic ablative RT (SABR) can overcome this through vascular targeting (12, 18–20). However, larger fraction (FX) sizes increase risk to surrounding renal parenchyma, given the kidney’s limited regenerative capacity and high FX sensitivity (21, 22). Dose-response relationship (DRR) analyses show an increase in nephrotoxicity with higher renal doses, with risk further amplified by larger FX (23–30). Reported modest long-term estimated glomerular filtration rate (eGFR) declines and low dialysis rates after kidney SABR largely reflect highly selected patients and optimized planning, limiting generalizability for defining safe incidental radiation doses to healthy renal tissue (15, 31–33). Despite advances in SABR enabling better parenchymal sparing, the long-term impact of incidental irradiation remains unclear (33, 34). A study on direct kidney irradiation for RCC showed RFD within the first year, stabilizing by year two, indicating early damage with a plateau phase (15). Given the limited kidney-specific SABR data, insights into the temporal pattern of radiation-induced kidney injury (RIKI) can be drawn from other irradiation settings involving kidney exposure. In retrospective analyses following total body irradiation, a study reported RFD beginning within approximately three months after treatment, whereas another described a plateau phase at around 26 months (28, 29). However, evidence is limited by small samples, heterogeneity in treatment and outcome definitions, hindering comparability and robust DRR analysis. This study evaluates the DRR of incidental kidney irradiation, with implications for kidney-sparing SABR concepts used in RCC, focusing on non-tumor-bearing renal parenchyma. We hypothesize that higher kidney dose is associated with RFD, as reflected by Common Terminology Criteria for Adverse Events (CTCAE)-graded CKD worsening (CTCAE v5.0; MedDRA v20.0). Our objectives are to characterise the DRR, assess the temporal pattern of RIKI, and identify clinical and dosimetric factors to inform safe kidney dose constraints in nephron-sparing SABR for primary RCC.

## 2 Materials and methods

### 2.1 Patients and data

The medical records and RT plans of patients treated at a German tertiary university cancer center between 01/2012 and 07/2024 were retrospectively analysed. Approval for this study and a waiver of written informed consent were granted by the institutional review board (ref. no. 10/5/22). Patients were identified if at least one kidney received a cumulative renal dose of ≥10 Gy, or if they underwent RT with a single-FX dose of >3 Gy, reflecting stereotactic treatment regimens (STX) (35). In all cases, renal irradiation occurred incidentally during RT of non-renal trunk targets. The screening cohort comprised 731 patients. Clinical data, laboratory values, nephrotoxic medications, comorbidities, and procedures potentially affecting renal function were extracted from electronic records. RT characteristics (dose, FX) were extracted from the Eclipse treatment planning system via the Eclipse Scripting API (ESAPI; Varian, Siemens Healthineers). Nephrotoxic medications were defined as NSAID use, platinum-based chemotherapy, or other recognized nephrotoxic agents and categorized by primary toxicity. Comorbidities were defined as pre-existing conditions, including hypertension, diabetes mellitus, cerebrovascular disease, cardiovascular disease, and acute kidney disease, and were grouped by organ system. The eGFR was calculated with the Chronic Kidney Disease Epidemiology Collaboration creatinine equation (CKD-EPI 2021) (36). The equivalent dose in 2 Gy FX (EQD2) was determined with the linear-quadratic model, assuming an α/β ratio of 3 for renal tissue, enabling standardized comparison across FXation schemes (21, 23). Patients with renal EQD2 <10 Gy were excluded. For bilateral exposure ≥10 Gy, EQD2_mean was the mean, EQD2_max the higher dose; for unilateral exposure, both referred to the irradiated kidney.

### 2.2 Renal function and endpoint definition

**Figure 1:**
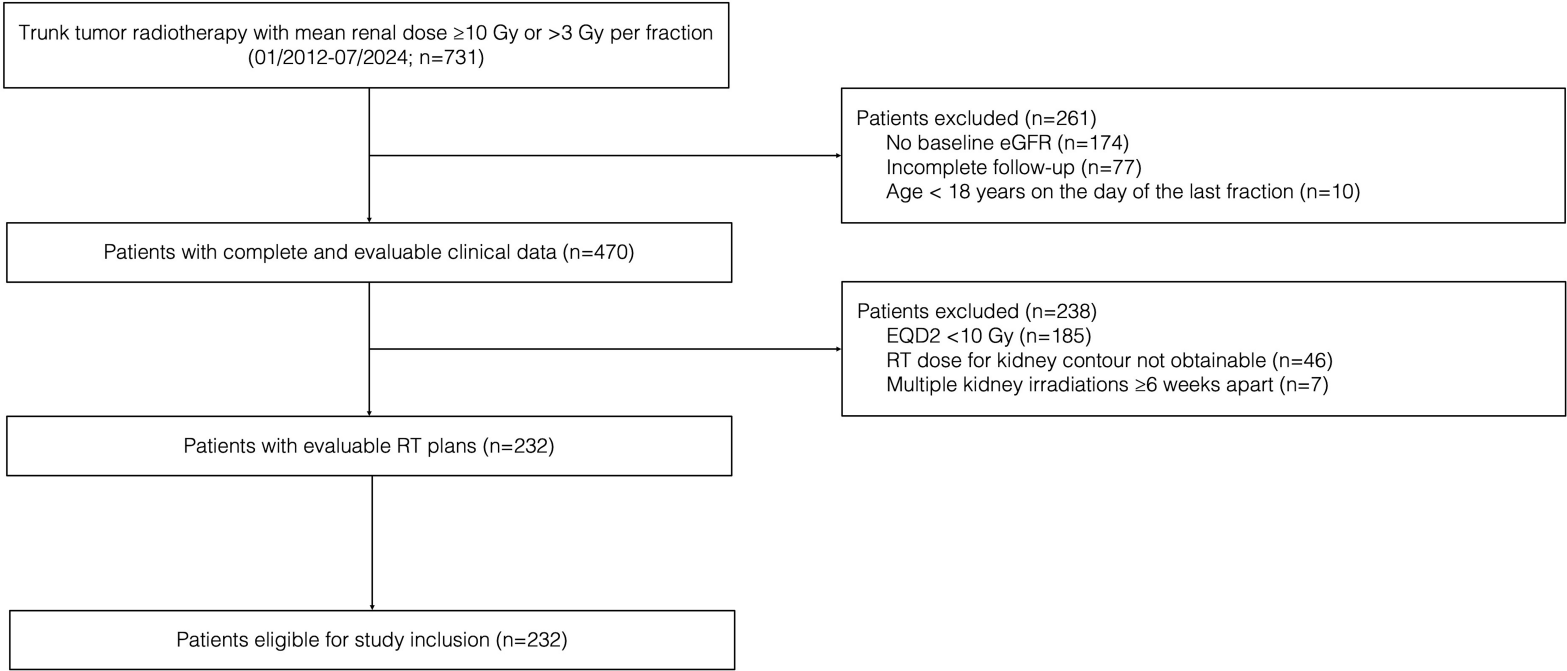
Patient screening.

Baseline eGFR was defined as the median of all measurements within one year before the last day of kidney-exposing RT. The eGFR was graded for CKD according to CTCAE v5.0 (MedDRA v20.0). The primary endpoint was defined as an increase of at least one CTCAE grade compared with baseline. To reduce the risk of misclassifying transient eGFR fluctuations as CKD worsening, an event was recorded only when two consecutive post-RT eGFR measurements both met the criteria for this higher CTCAE grade. The event date was assigned to the second of these two measurements. Patients lacking baseline eGFR or ≥2 post-RT measurements, or not meeting eligibility criteria, were excluded (**Figure 1**), resulting in a final cohort of 232 patients. No imputation of missing data was performed. Baseline characteristics and dose parameters are shown in **Table 1**. Data preprocessing was performed using Python and Microsoft Excel.

**Table 1:** Patient and treatment characteristics.

Variable	Value
Age	64 years (SD 12.22)
Sex	
Male	102 patients (43,97%)
Female	130 patients (56,03%)
Number of kidneys exposed	
One	120 patients (51.72%)
Two	112 patients (48.28%)
Radiotherapy indication	
Secondary malignant neoplasm of bone and bone marrow (ICD-10: C79.5)	42 patients (18.1%)
Malignant neoplasm: Pancreas, unspecified (ICD-10: C25.9)	27 patients (11.64%)
Malignant neoplasm: Oesophagus, unspecified (ICD-10: C15.9)	13 patients (5.6%)
Radiotherapy data	
Normofractionated	219 patients (94.4%)
STX	13 patients (5.60%)
Mean kidney dose (EQD2_mean)	14.2 Gy (SD 4.68 Gy)
Mean kidney dose (EQD2_max)	14.6 Gy (SD 4.86 Gy)
Curative (>20 fractions)	120 patients (51.72%)
Palliative (≤20 fractions)	112 patients (48.28%)
Locally curative (single fraction dose >3 Gy)	13 patients (5.60%)
Event rate	109 patients (46.98%)
Median follow-up, total cohort	170 days (IQR 60-541)
Median Time to Event	173 days (IQR 55-423)
Median Follow-Up, censored patients	168 days (IQR 66-618)
Baseline kidney function (CKD, CTCAE)	
Grade 0	135 patients (58.19%)
Grade 1	74 patients (31.9%)
Grade 2	19 patients (8.19%)
Grade 3	2 patients (0.86%)
Grade 4	2 patients (0.86%)
Kidney function after radiotherapy	
Grade 0	113 patients (48.71%)
Grade 1	53 patients (22.84%)
Grade 2	50 patients (21.55%)
Grade 3	11 patients (4.74%)
Grade 4	5 patients (2.16%)
Medication	
Prerenal	1 (0.43%)
Tubular	12 (5.17%)
Glomerular	0
Immune-mediated	56 (24.14%)
Comorbidity	
Kidney	6 (2.58%)
Heart	15 (6.47%)
Hypertension	38 (16.38%)
Diabetes mellitus	17 (7.32%)
CNS	4 (1.72%)

### 2.3 Statistical methods

Baseline characteristics were summarized using descriptive statistics. Continuous variables are reported as mean with standard deviation (SD) or median with interquartile range (IQR), and categorical variables as n and %. Time-to-event analyses used survival methods, with follow-up from last FX to event or last follow-up. Maximally selected log-rank statistics identified data-driven cut-offs; Kaplan-Meier curves were generated for resulting risk groups. Dose was modeled continuously using restricted cubic splines (3 knots) in Cox proportional hazards regression. Primary analyses used multivariable Cox proportional hazards regression adjusted for age, sex, baseline eGFR, nephrotoxic medication, comorbidity, FX, and dose per FX. Univariable analyses and correlation assessments (Pearson’s, Spearman’s, or Cramér’s V) guided covariate selection. Adjusted HRs with 95% CIs and two-sided p-values (α=0.05) were reported. Statistical analyses were conducted separately for EQD2_mean and EQD2_max. Analyses were performed in R using base R functions and the packages readxl, dplyr, tidyr, lubridate, stringr, purrr, tibble, DescTools, survival, survminer, rms, glmmTMB, broom, broom.mixed, ggplot2, and openxlsx.

## 3 Results

Of 232 eligible patients, 109 (46.98%) met the primary endpoint of CTCAE-graded CKD deterioration, including 68 (29.31%) with new-onset deterioration. During a median follow-up of 170 days (IQR 60-541), the median time to deterioration was 5.7 months among patients who experienced an event (173 days; IQR 55-423), while censored patients had a median follow-up of 5.5 months (168 days; IQR 66-618), providing temporal context for time-to-event analyses performed in this cohort. Post-RT, CTCAE renal toxicity was grade 0 in 113 patients (48.71%), grade 1 in 53 (22.84%), grade 2 in 50 (21.55%), grade 3 in 11 (4.74%), and grade 4 in 5 (2.16%). Overall, CTCAE grade ≥3 toxicity occurred in 16/232 patients (6.90%); among those with baseline CTCAE grade 1-2 (n=93), progression to CTCAE grade ≥3 was observed in 14 (15.1%).

Radiotherapy exposure and kidney dose: Most patients received normofractionated RT (219/232, 94.40%), whereas 13/232 (5.60%) received STX; these 13 cases also constituted locally curative regimens with a single-FX dose >3 Gy (5.6%). By FX, 120/232 treatment courses (51.72%) were classified as curative (>20 fractions) and 112/232 (48.28%) as palliative (≤20 fractions). Bilateral kidney exposure occurred in 112 patients (48.28%). Mean renal doses were EQD2_mean 14.2 Gy (SD 4.68 Gy) and EQD2_max 14.6 Gy (SD 4.86 Gy), and dose-response analyses were performed separately for each metric.

**Figure 2:**
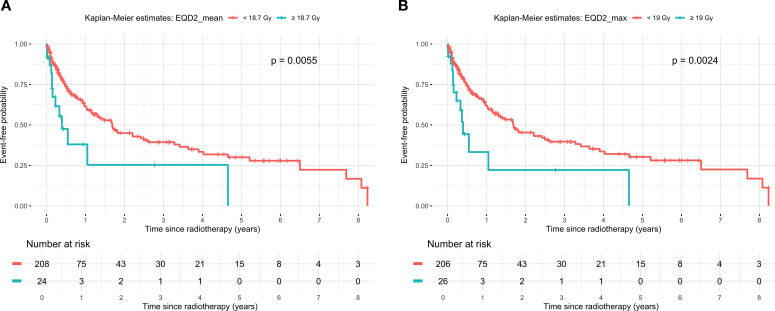
**(A)** Kaplan-Meier estimates by EQD2_mean. **(B)** Kaplan-Meier estimates by EQD2_max.

**Figure 3:**
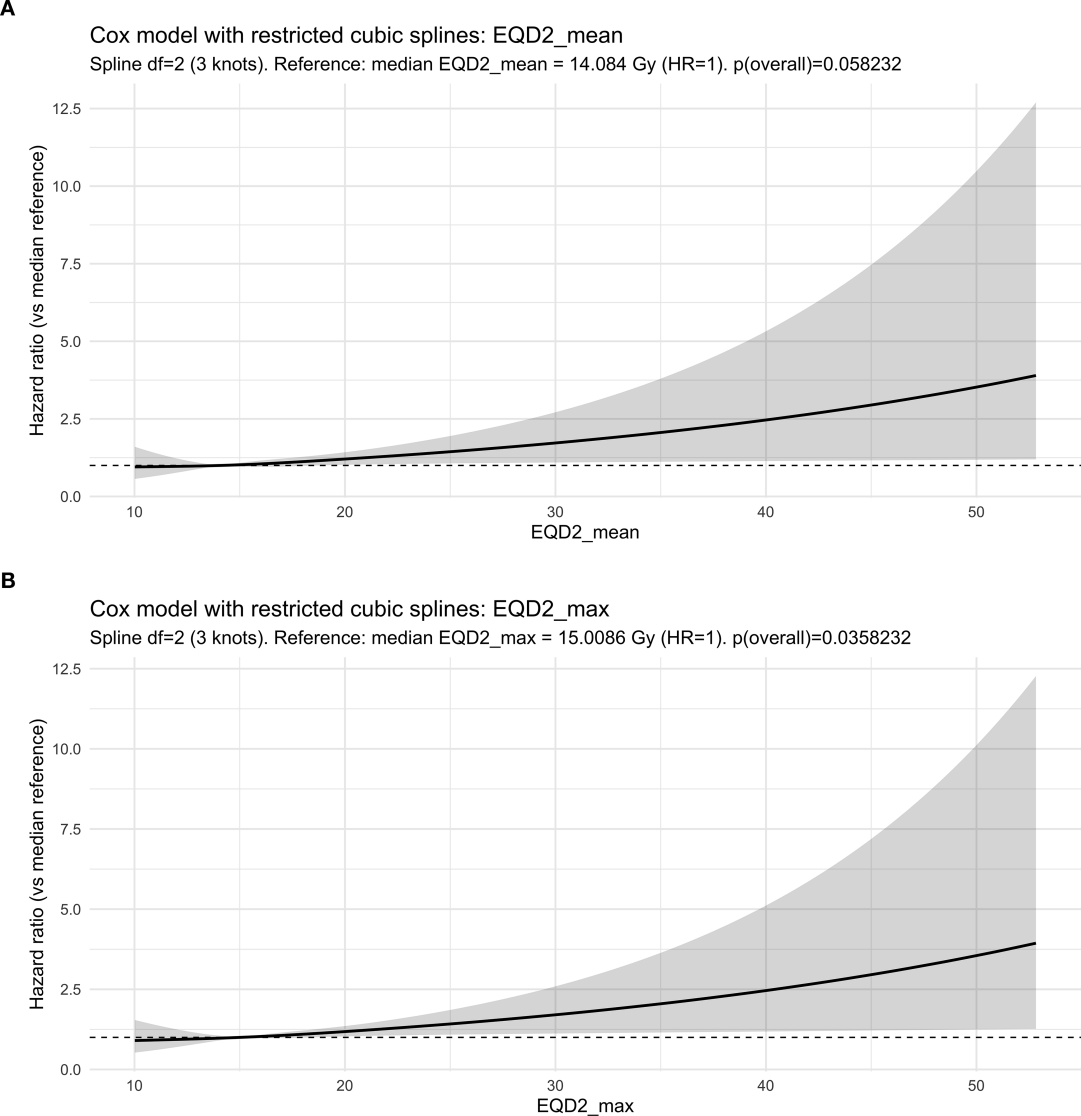
**(A)** Restricted cubic spline analysis of EQD2_mean. **(B)** Restricted cubic spline analysis of EQD2_max.

Dose-response by risk stratification and continuous modeling: Using maximally selected log-rank statistics, we identified dose levels for increased CKD deterioration risk. For EQD2_mean, the data-driven threshold was 18.7 Gy: patients receiving ≥18.7 Gy (n=24) had a higher risk of deterioration than those receiving <18.7 Gy (n=208) (HR 2.25, 95% CI 1.25-4.05; log-rank p=0.005; **Figure 2A**). Similarly, for EQD2_max, a threshold of 19 Gy was observed: patients receiving ≥19 Gy (n=26) had a higher risk than those receiving <19 Gy (n=206) (HR 2.37, 95% CI 1.34-4.20; log-rank p=0.002; **Figure 2B**). To evaluate the DRR across the full dose range (rather than at a single cut-off), we fitted restricted cubic spline univariable Cox models (three knots), expressing hazard ratios relative to the median dose (HR = 1 at the median). In these models, the overall association with deterioration was p=0.0580 for EQD2_mean (**Figure 3A**) and p=0.0358 for EQD2_max (**Figure 3B**), with no evidence of non-linearity (EQD2_mean p=0.7441; EQD2_max p=0.7976). Taken together, both the threshold-based and continuous analyses supported a consistent dose-response signal across dose metrics, justifying inclusion of EQD2_mean and EQD2_max in multivariable models.

**Figure 4:**
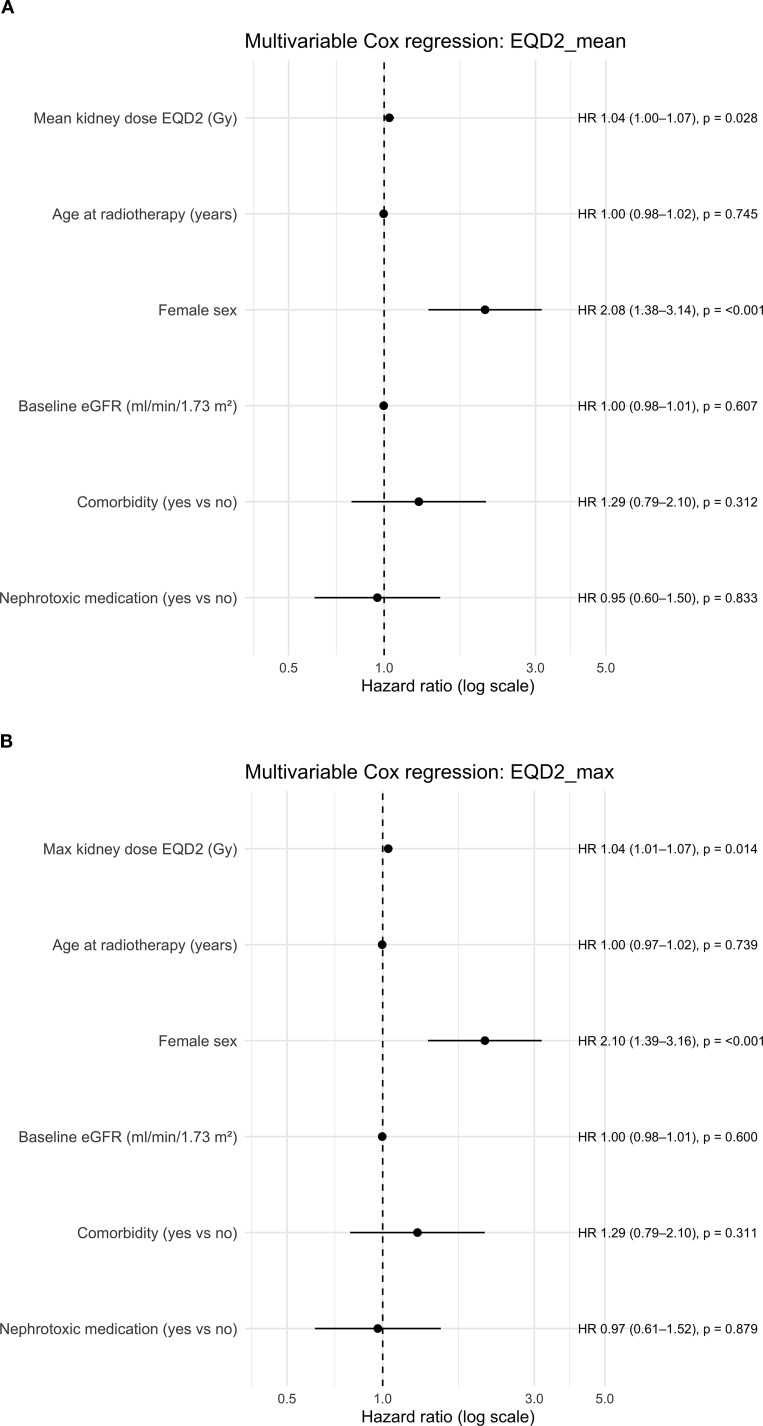
**(A)** Multivariable Cox regression analysis of renal function deterioration for EQD2_mean. **(B)** Multivariable Cox regression analysis of renal function deterioration for EQD2_max.

Predictors in univariable and multivariable Cox regression: On univariable analysis, higher EQD2_mean (HR 1.03, 95% CI 1.01-1.06; p=0.021), higher EQD2_max (HR 1.04, 95% CI 1.01-1.06; p=0.012), and female sex (HR 2.16, 95% CI 1.44-3.25; p<0.001) were significantly associated with CKD deterioration. EQD2_mean and EQD2_max were highly correlated (Pearson’s r=0.98; Spearman’s ρ=0.98); baseline eGFR and age showed inverse correlations (Pearson’s r=-0.53; Spearman’s ρ=-0.62), and no relevant associations between categorical variables were observed using Cramér’s V. In multivariable Cox models adjusting for age, sex, baseline eGFR, nephrotoxic medication, comorbidity, FX and dose per FX, renal dose remained independently associated with the endpoint (EQD2_mean: HR 1.04, 95% CI 1.00-1.07; p=0.028; EQD2_max: HR 1.04, 95% CI 1.01-1.07; p=0.014). Female sex also remained independently associated with higher risk (EQD2_mean model: HR 2.08, 95% CI 1.38-3.14; p<0.001; EQD2_max model: HR 2.10, 95% CI 1.39-3.16; p<0.001); age, baseline eGFR, nephrotoxic medication, comorbidity, FX, and dose per FX were not statistically significant. See **Table 2** and **Figures 4A, B** for adjusted estimates. Additional analyses did not identify an evident measured confounder explaining the association between female sex and renal function deterioration (**Supplementary Table 5**).

**Table 2:** Multivariable Cox regression results.

Variable	HR	CI low	CI high	p-value
a Results for EQD2_mean				
EQD2_mean	1.04	1.00	1.07	0.028
Age	1	0.98	1.02	0.717
Female Sex	2.08	1.38	3.14	<0.001
Baseline eGFR	1	0.98	1	0.596
Medication	0.95	0.6	1.5	0.785
Comorbidity	1.29	0.79	2.1	0.304
Number of fractions	1.01	0.99	1.03	0.208
Dose per fraction	1.01	0.88	1.17	0.853
b Results for EQD2_max				
EQD2_max	1.04	1.01	1.07	0.014
Age	1	0.97	1.02	0.739
Female Sex	2.1	1.39	3.16	<0.001
Baseline eGFR	1	0.98	1.01	0.56
Medication	0.97	0.61	1.52	0.879
Comorbidity	1.29	0.79	2.1	0.311
Number of fractions	1.01	0.99	1.03	0.192
Dose per fraction	1.01	0.88	1.16	0.852

## 4 Discussion

Higher kidney EQD2_mean and EQD2_max were independently associated with increased CKD risk after adjustment for age, sex, baseline renal function, comorbidities, medication use, FX, and dose per FX. HRs were 1.04 per 1 Gy increase (EQD2_mean: 95% CI 1.00-1.07; EQD2_max: 95% CI 1.01-1.07). This demonstrates a DRR even within a low-to- moderate dose range, with mean kidney EQD2_mean and EQD2_max values of 14.2 Gy (SD 4.68) and 14.6 Gy (SD 4.86), respectively. Beyond dose, female sex emerged as a relevant risk factor (EQD2_mean: HR 2.1; 95% CI 1.38-3.14; p<0.001; EQD2_max: HR 2.10; 95% CI 1.40-3.16; p<0.001), indicating that risk is not determined by dosimetry alone. As additional analyses did not identify an evident explanation for this association in our dataset, the finding should be interpreted with caution and regarded as hypothesis-generating. Potential methodological contributors include sex-specific coefficients in creatinine-based eGFR equations, creatinine-related limitations due to differences in muscle mass, and absolute CTCAE eGFR thresholds (36, 37). These factors may reduce the distance to the next toxicity grade and contribute to the observed association in women. However, this remains speculative and does not exclude biological, clinical, or treatment-related mechanisms. Further studies are needed to clarify this finding. Spline-based univariable Cox models suggested a monotonic increase in risk: EQD2_max showed a significant overall association (p=0.0358) and EQD2_mean a borderline association (p=0.0580), while non-linear components were non-significant for both (EQD2_mean: p=0.7441; EQD2_max: p=0.7976). Thus, within this dose range, risk increased linearly without a clear threshold or curvature. Nevertheless, data-driven cut-offs of 18.7 Gy (EQD2_mean) and 19 Gy (EQD2_max) were identified, defining small high-dose subgroups (n=24/208 and n=26/206, respectively). Due to unbalanced group sizes, the cut-offs should be interpreted exploratory. However, the low number of high-dose patients reflects kidney-sparing treatment planning, and the identified dose thresholds align with RIKI literature, supporting their clinical relevance. Several studies have reported a DRR for kidney irradiation, with toxicity increasing by 10-20% per 1–2 Gy and decreasing with more FX regimens at comparable total dose (27, 28, 30, 38). Dose-volume tolerance data for bilaterally irradiated kidneys indicate an approximate 5% risk of clinically relevant dysfunction at mean doses of 15–23 Gy and about 50% at 28 Gy (23–26). Our cut-offs fall within this range, indicating that even small dose increases are clinically relevant for RFD. This is consistent with the growing survivorship-focused perspective that non-cancer morbidity and mortality represent important long-term endpoints in cancer patients, highlighting the clinical value of identifying and minimizing treatment-related organ toxicity (39–42). Furthermore, the median time to event was 173 days (IQR 55–423 days), which is consistent within the previously reported time frame of 3–24 months for radiation-associated RFD (15, 28, 29).

### 4.1 Implications for SABR concepts in primary RCC

Kidneys were not the primary RT target in our cohort; exposure resulted from scatter and penumbra of adjacent targets, with few patients receiving high renal doses. This mirrors kidney-sparing SABR for primary RCC, where a small volume receives ablative doses, while most of the kidney remains in a low-to-moderate dose range. As SABR gains acceptance for inoperable RCC with high local control, our findings on renal DRR in a low-to-moderate range offer insights into functional tolerance of non-target kidney tissue (15, 18–20). Although our cohort did not receive kidney-directed SABR, the findings may inform dose constraints and treatment planning in emerging SABR approaches for RCC. Furthermore, we observed no difference in early RFD between patients with one versus two exposed kidneys, consistent with published SABR series in primary RCC (31). This may reflect effective renal sparing and compensatory renal function (5, 28). Previous studies have identified reduced baseline eGFR as risk factor for renal deterioration after RT (31, 32). In our cohort, however, baseline eGFR was not associated with the endpoint in multivariable models for either EQD2_mean or EQD2_max. Accordingly, although 46.98% of patients experienced CTCAE-graded RFD, most events were mild (grades 1-2: 40.09%), while clinically relevant toxicity was rare (grade 3: 4.74%; grade 4: 2.16%). These findings align with SABR series in primary RCC, showing modest long-term eGFR decline and low dialysis rates (15, 31–33). These findings, from predominantly elderly cohorts, may underrepresent outcomes in fitter patients, although this requires prospective validation. Taken together, existing SABR data and our results support a dose dependent model of RIKI that extends from high-dose tumor treatment volumes to the surrounding healthy kidney tissue.

#### 4.1.1 Benchmark against surgery and clinical implications

Surgical series report variable chronic renal outcomes owing to differing endpoints. Within the first postoperative year, about 20-30% are reported to progress to CKD (7, 8). Other studies report higher rates when using the combined endpoint of new-onset or progressive CKD, with worse outcomes after radical compared with PN (56% vs 44%) (11). In addition, after PN, 29% of patients with baseline CKD stage 1–2 progressed to stage ≥3 during follow-up (10). Against this background, our data indicate that RFD after RT is common, yet severe toxicity is rare. Any CKD deterioration occurred in 46.98%, including 29.31% with new-onset deterioration. Clinically consequential events were rare: CTCAE grade ≥3 toxicity occurred in 6.90%, and grade 4 toxicity occurred in 2.16%. Among patients with baseline CTCAE grade 1-2, progression to CTCAE grade ≥3 occurred in 15.1%. Despite cross-modality limitations, data suggest comparable renal risk between surgery and RT in RCC, supporting SABR as a nephron-sparing option for inoperable patients, when incidental dose to healthy renal parenchyma is minimized. Our findings inform the development of safe SABR protocols for primary RCC: First, dose to the non-tumor-bearing renal parenchyma should be kept as low as reasonably achievable, as even moderate increases were associated with RFD. Second, women may be more vulnerable to RIKI and may benefit from stricter dose constraints and closer post-treatment monitoring. Third, our data provide a quantitative reference for future SABR studies aiming to define kidney dose limits associated with RFD comparable to those observed after nephrectomy.

### 4.2 Strenghts and limitations

Strengths of this study include its focus on low-to-moderate kidney irradiation – a common scenario when preserving non-tumor-bearing renal parenchyma is prioritized. Detailed RT data enabled assessment of dosimetric and FX factors, while longitudinal laboratory follow-up enabled robust evaluation of RFD using CTCAE-graded CKD worsening as a standardized, clinically relevant endpoint with multivariable adjustment.

Limitations include the retrospective single-centre design, potential confounding, treatment heterogeneity, and the rarity of SABR treatments rendering dose constraints exploratory. Although EQD2 conversion used the linear-quadratic model with an α/β ratio of 3 Gy, extrapolation to SABR should be interpreted cautiously because this model has limitations in the ablative dose range. Given the small stereotactic subgroup in our cohort, the observed DRR should be considered biologically supportive but not directly transferable to renal SABR without dedicated validation. Competing events such as death before renal function deterioration could not be formally accounted for due to incomplete mortality data inherent to the retrospective study design. Therefore, cumulative incidence of renal toxicity may potentially be overestimated. Nevertheless, the findings provide important insight into renal DRR and warrant confirmation in prospective multicentre studies.

## 5 Conclusions

Our study identifies higher kidney dose as an independent predictor of CTCAE-graded CKD worsening, demonstrating a clinically relevant DRR in the low-to-moderate dose range applicable to nephron-sparing SABR for primary RCC. Renal injury occurred within months after treatment and severe toxicity remained uncommon. Female sex was associated with a higher risk of RFD. Overall, renal outcomes were clinically acceptable, supporting RT as a safe option for RCC in selected patients and underscoring the importance of minimizing dose to non-tumor-bearing renal parenchyma. Future research should prospectively validate these associations, optimize treatment planning, and refine kidney dose constraints.

## Data Availability

The original contributions presented in the study are included in the article/**Supplementary Material**, Further inquiries can be directed to the corresponding author.
