# Supplementary material for: Post-treatment renal function deterioration following radiation therapy: implications for SABR in primary renal cell cancer
Source: Front Oncol. 2026 Jun 23;16:1868006. doi: 10.3389/fonc.2026.1868006 (PMC13337510; doi:10.3389/fonc.2026.1868006)
Supplement: Supplementary file 1 [file Table1.docx]

Supplementary Material

1. Univariate Cox regression results

| **Category** | **Variable** | **HR** | **95% CI** | **P value** | **Note** |
| --- | --- | --- | --- | --- | --- |
| Significant results | Sex | 2.16 | 1.44 – 3.25 | <0.001 | Standard |
| Significant results | EQD2 mean | 1.03 | 1.01 – 1.06 | 0.021 | Standard |
| Significant results | EQD2 max | 1.03 | 1.01 – 1.06 | 0.012 | Standard |
| Significant results | Diagnosis: C53.9 | 3.88 | 1.94 – 7.78 | <0.001 | Ridge (theta=0.5) |
| Significant results | Baseline CTCAE grade 3 | 6.21 | 1.50 – 25.70 | 0.012 | Standard |
| Radiation factors | Dose/fraction (Gy) | 1.03 | 0.93 – 1.15 | 0.541 | Standard |
| Radiation factors | EQD2 difference | 1.04 | 0.99 – 1.11 | 0.141 | Standard |
| Radiation factors | Number of fractions | 1.01 | 0.99 – 1.02 | 0.297 | Standard |
| Radiation factors | Type | 0.81 | 0.36 – 1.86 | 0.628 | Standard |
| Radiation factors | N exposed | 1.00 | 0.68 – 1.46 | 0.991 | Standard |
| Age | Age at radiotherapy | 1.00 | 0.99 – 1.02 | 0.709 | Standard (HR per year) |
| Age | Age at radiotherapy | 1.03 | 0.89 – 1.20 | 0.709 | Standard (HR per 10 years) |
| Baseline | Baseline median eGFR | 1.09 | 0.90 – 1.31 | 0.394 | Standard (HR per 1 SD) |
| Baseline | Baseline CTCAE grade | 0.86 | 0.64 – 1.16 | 0.327 | Standard (HR per grade increase) |
| Baseline | Baseline CTCAE grade 1 | 0.77 | 0.51 – 1.17 | 0.216 | Standard |
| Baseline | Baseline CTCAE grade 2 | 0.59 | 0.24 – 1.47 | 0.258 | Standard |
| Baseline | Baseline CTCAE grade 3 | 6.21 | 1.50 – 25.70 | 0.012 | Standard |
| Baseline | Baseline CTCAE grade 4 | 0.00 | 0.00 – NA | 0.996 | Standard |
| Comorbidities | Kidney | 1.38 | 0.74 – 2.59 | 0.308 | Standard |
| Comorbidities | CNS | 0.64 | 0.23 – 1.73 | 0.374 | Standard |
| Comorbidities | Coronary heart disease | 0.81 | 0.50 – 1.31 | 0.392 | Standard |
| Comorbidities | Diabetes | 1.20 | 0.77 – 1.88 | 0.421 | Standard |
| Comorbidities | Hypertension | 1.15 | 0.78 – 1.69 | 0.484 | Standard |
| Comorbidities | Any | 1.13 | 0.75 – 1.71 | 0.560 | Standard |
| Procedures | 8-54 | 1.20 | 0.81 – 1.77 | 0.368 | Standard |
| Procedures | 8-85 | 1.51 | 0.37 – 6.16 | 0.563 | Standard |
| Procedures | 8-13 | 0.93 | 0.40 – 2.13 | 0.857 | Standard |
| Procedures | 1-46 | 0.00 | 0.00 – NA | 0.994 | Ridge (theta=0.5) |
| Procedures | 5-55 | NA | NA | NA | Too little data |
| Medications | Tubular | 0.77 | 0.34 – 1.78 | 0.550 | Standard |
| Medications | Immunological | 0.94 | 0.60 – 1.47 | 0.780 | Standard |
| Medications | Any | 0.89 | 0.57 – 1.38 | 0.600 | Standard |

2. Pearson correlation matrix of variables included in the multivariable analysis

| **Variable** | **Age** | **EQD2_mean** | **EQD2_max** | **Baseline Median eGFR** | **Medication** | **Comorbidity** | **Anz Number of fractions** | **Dose/Fraction** |
| --- | --- | --- | --- | --- | --- | --- | --- | --- |
| **Age** | 1 | 0.01 | 0.01 | -0.53 | 0.07 | 0.35 | -0.09 | -0.02 |
| **EQD2_mean** | 0.01 | 1 | 0.98 | -0.16 | -0.01 | -0.02 | -0.18 | 0.36 |
| **EQD2_max** | 0.01 | 0.98 | 1 | -0.15 | -0.03 | -0.02 | -0.17 | 0.33 |
| **Baseline Median eGFR** | -0.53 | -0.16 | -0.15 | 1 | 0.05 | -0.29 | 0.17 | -0.04 |
| **Medication** | 0.07 | -0.01 | -0.03 | 0.06 | 1 | 0.15 | 0.01 | -6.48E-05 |
| **Comorbidity** | 0.35 | -0.02 | -0.02 | -0.29 | 0.15 | 1 | -0.05 | -0.11 |
| **Number of fractions** | -0.09 | -0.18 | -0.17 | 0.17 | 0.01 | -0.05 | 1 | -0.46 |
| **Dose/Fraction** | -0.02 | 0.36 | 0.33 | -0.04 | -6.48E-05 | -0.11 | -0.46 | 1 |

3. Spearman correlation matrix of variables included in the multivariable analysis

| **Variable** | **Age** | **EQD2_mean** | **EQD2_max** | **Baseline Median eGFR** | **Medication** | **Comorbidity** | **Anz Number of fractions** | **Dose/Fraction** |
| --- | --- | --- | --- | --- | --- | --- | --- | --- |
| **Age** | 1 | -0.01 | -0.02 | -0.62 | 0.06 | 0.32 | -0.02 | -0.01 |
| **EQD2_mean** | -0.01 | 1 | 0.98 | -0.06 | -0.11 | -0.02 | -0.13 | 0.18 |
| **EQD2_max** | -0.02 | 0.98 | 1 | -0.06 | -0.13 | -0.03 | -0.11 | 0.15 |
| **Baseline Median eGFR** | -0.62 | -0.06 | -0.06 | 1 | 0.03 | -0.3 | 0.14 | -0.13 |
| **Medication** | 0.06 | -0.11 | -0.13 | 0.03 | 1 | 0.15 | 0.04 | 0.04 |
| **Comorbidity** | 0.32 | -0.02 | -0.03 | -0.3 | 0.15 | 1 | -0.02 | -0.02 |
| **Number of fractions** | -0.02 | -0.13 | -0.11 | 0.14 | 0.04 | -0.02 | 1 | -0.6 |
| **Dose/Fraction** | -0.01 | 0.18 | 0.15 | -0.13 | 0.04 | -0.02 | -0.6 | 1 |

4. Cramér’s V correlation matrix of variables included in the multivariable analysis

| **var1** | **var2** | **Cramér’s V** |
| --- | --- | --- |
| Medication | Comorbidity | 0.15 |
| Sex | Medication | 0.04 |
| Sex | Comorbidity | 0,11 |

5. Potential confounders of the association between female sex and renal function deterioration

| **Analysis** | **Result** |
| --- | --- |
| **Primary association: female sex and renal function deterioration** | HR 2.08, 95% CI 1.38-3.14, p<0.001 |
| **Exclusion of cervical cancer cases (C53.9)** | HR 1.96, 95% CI 1.29-2.96, p=0.002 |
| **Restriction to diagnoses represented in both sexes** | HR 2.01, 95% CI 1.25-3.24, p=0.004 |
| **Platinum-based medication during follow-up** | No female patient received platinum-based medication during the observation period. |
| **Bilaterally exposed kidneys** | Male: 44.1% vs. female: 51.5%, RR 1.17, 95% CI 0.89-1.54, p=0.262 |
| **Baseline eGFR** | Male: median 95.8 ml/min/1.73 m² (IQR 84.8-106.0) vs. female: median 91.9 ml/min/1.73 m² (IQR 75.3-101.0), p=0.044 |
| **EQD2_mean** | Male: median 14.8 Gy (IQR 12.1-16.4) vs. female: median 14.0 Gy (IQR 11.9-16.0), p=0.458 |
| **Nephrotoxic medication** | Male: 26.5% vs. female: 23.1%, RR 0.87, 95% CI 0.56-1.37, p=0.551 |
